# Molecular Cloning, Bioinformatic Analysis, and Expression of* Bombyx mori* Lebocin 5 Gene Related to* Beauveria bassiana* Infection

**DOI:** 10.1155/2017/9390803

**Published:** 2017-01-17

**Authors:** Dingding Lü, Chengxiang Hou, Guangxing Qin, Kun Gao, Tian Chen, Xijie Guo

**Affiliations:** ^1^School of Biotechnology and Sericultural Research Institute, Jiangsu University of Science and Technology, Zhenjiang 212018, China; ^2^Nursing School, Zhenjiang College, Zhenjiang 212003, China

## Abstract

A full-length cDNA of lebocin 5 (BmLeb5) was first cloned from silkworm,* Bombyx mori*, by rapid amplification of cDNA ends. The BmLeb5 gene is 808 bp in length and the open reading frame encodes a 179-amino acid hydroxyproline-rich peptide. Bioinformatic analysis results showed that BmLeb5 owns an O-glycosylation site and four RXXR motifs as other lebocins. Sequence similarity and phylogenic analysis results indicated that lebocins form a multiple gene family in silkworm as cecropins. Quantitative real-time PCR analysis revealed that BmLeb5 was highest expressed in the fat body. In the silkworm larvae infected by* Beauveria bassiana*, the expression level of BmLeb5 was upregulated in the fat body and hemolymph which are the most important immune tissues in silkworm. The recombinant protein of BmLeb5 was for the first time successfully expressed with prokaryotic expression system and purified. There are no reports so far that the expression of lebocins could be induced by entomopathogenic fungus. Our study suggested that BmLeb5 might play an important role in the immune response of silkworm to defend* B. bassiana* infection. The results also provided helpful information for further studying the lebocin family functioned in antifungal immune response in the silkworm.

## 1. Introduction

Silkworm (*Bombyx mori*) is one of the important economic insects spinning cocoon. But the silkworm always suffers from diseases in the sericulture production, especially white muscardine caused by infection with entomopathogenic fungi.* Beauveria bassiana *was the first discovered fungus causing the devastating white muscardine disease to the silkworms and is always a troublesome pathogenic fungus in sericulture [1–3].* B. bassiana* works by direct penetrating into the insect cuticle and colonize* in vivo* [4]. Then the fungi fight with the host's innate immune defense, consisting mainly of cellular and humoral mechanisms [5, 6]. A set of antimicrobial peptides (AMPs) are produced to protect the host against invading microbes in humoral immune system [7]. Insect AMPs are mainly synthesized in the fat body, secreted into the hemolymph, and regulated by Rel family transcription factors via activating the Toll and IMD signaling pathways [8–12].

In the silkworm, the AMPs have been identified into six groups: cecropins [13–15], moricins [16], gloverins [17], attacins [18], enbocins [19], and lebocins [20, 21]. Lebocins are first isolated from silkworm and classified as proline-rich AMPs [20, 22]. Then lebocins also have been identified in* Trichoplusia ni*,* Samia cynthia ricini*,* Pseudoplusia includes*, and* Manduca sexta* [23–26]. Lebocins are generally synthesized in the form of precursors and usually inactive and need to be proteolytically processed at RXXR motifs to generate short active peptides [27]. Unlike the fact that* M. sexta* active lebocin peptides are located at the N-terminal ends,* B. mori* lebocins are close to the C-terminal with 32 residue peptides. These peptides are O-glycosylated at Thr^15^ which is important for the antibacterial activity [22]. But abaecin of the honeybee, whose primary structure and antimicrobial activity resemble lebocins, is not O-glycosylated [28].

Four lebocins 1–4 were isolated from silkworm larvae immunized with* Escherichia coli*. Lebocins 1 and 2 have the same amino acid sequences and consequently named lebocin 1/2. Lebocin 1/2 occurred tissues-specifically in the fat body of silkworm after bacterial induction [20]. Lebocins 3 and 4 could be induced by lipopolysaccharide (LPS) and represent tissues-specifically in the fat body and hemocytes [21]. Lebocin 1–3 showed lower antimicrobial activity against Gram-negative bacteria than cecropin B (an AMP of silkworm) in physiological condition. But they exhibited a higher bactericidal activity against* E. coli *under low-ionic-strength [22]. The self-defense mechanism of these lebocins was not clear. Lebocin 3 was confirmed that it has a synergistic effect with cecropin D in antibacterial activity [29]. Meanwhile, there are reports that silkworm lebocins could be induced by LPS or* E. coli*, but there are no reports that fungus could induce their expression and they could also not be expressed in vitro so far.

In this study, a new upregulated homologous sequences gene of lebocins was derived from our transcriptome analysis to compare differently expressed genes in the silkworm between* B. bassiana*-infected and normal larvae [30]. In order to elucidate the expression mechanisms of lebocin, the full-length cDNA of BmLeb5 by RACE according to the homologous sequences was cloned. Quantitative real-time PCR was used to analyze the expression characteristics of* BmLeb5* in the whole larvae, in the fat body, and in hemolymph at different periods after* B. bassiana *infection. The recombinant protein of BmLeb5 was successfully expressed with prokaryotic expression system and purified.

## 2. Materials and Methods

### 2.1. Silkworm and Fungal Strains

In this study, the experimental silkworm larvae were p50 strain (provided by Sericultural Research Institute of Chinese Academy of Agricultural Sciences) which were fed with fresh mulberry leaves under a photoperiod of 12 h light and 12 h dark at 25°C. The larvae were raised to third or fifth instar for subsequent experiments. Strain BbHN6 of* B. bassiana*, originally isolated from the cadaver of muscardine silkworm, was also provided by Sericultural Research Institute of Chinese Academy of Agricultural Sciences as we reported before [31]. The conidia of* B. bassiana* were gathered by culturing the fungus on potato dextrose agar (PDA) for 14 days at 25 ± 1°C.

### 2.2. *B. bassiana* Inoculation

The concentration of conidia of BbHN6 was adjusted to 1 × 10^8^ conidia mL^−1^ in distilled water containing 0.01% (vol vol^−1^) Tween-20 after getting rid of mycelia. About 100 larvae of third or 300 larvae of fifth instar silkworm larvae were immersed in conidia suspension for 20 s and distributed into groups in a relative humidity of breeding environment for 48 hours (hrs) and afterward at ambient humidity. The larvae of control groups were treated in distilled water containing 0.01% (vol vol^−1^) Tween-20 and placed in the same breeding environment as the BbHN6-infected groups.

### 2.3. Tissues Collection

The tissues (cuticle, fat body, hemolymph, Malpighian tubule, midgut, and silk gland) of the fifth instar silkworm were collected from both control and BbHN6-infected groups from 8 hours after inoculation (hpi) to 54 hpi at intervals of four hours. The anatomical larvae tissues were quickly put into distilled water containing 0.15 M NaCl to remove attached mulberry leaf pieces. Then the same tissues of 10 larvae were pooled in a storage tube with 0.5 mL Trizol reagent (TaKaRa) and immediately frozen in liquid nitrogen. But the hemolymph was directly inpoured into the storage tubes from a ruptured proleg. All the samples were stored at −80°C for the following study. There were three repeated samples for every treatment.

### 2.4. Cloning of* BmLeb5* Gene

The extraction of total RNA was performed from third instar silkworm larvae using Trizol reagent (TaKaRa) and detected for the ratio of 28S : 18S by agarose electrophoresis. The open reading frame (ORF) was cloned from the third instar larvae by RT-PCR using the primers Leb5orfF (ATGTACAAGTTTTTAGTATTCAGT) and Leb5orfR (CTATTCTTGGAAAATATCCCTCGG) according to our transcriptome analysis and its homologous gene* Lebocin1/2* (NP_001037468.1). Full-length cDNA sequences of* BmLeb5 *were obtained using SMARTer® RACE 5′/3′ Kit (Clontech) according to the instructions of the manufacturer. The specific 5′RACE and 3′RACE primers were designed according to ORF of BmLeb5. GSP1 (GATTACGCCAAGCTTGGTCCCTTGTGTTACGGTGGCTCT) was used for cloning the 5′-untranslated regions (UTR). GSP2 (GATTACGCCAAGCTTAGAGCCACCGTAACACAAGGGACC) was used to amplify the 3′-UTR. The PCR program was conducted using the following parameters: denaturation at 94°C for 5 min, followed by 25 cycles of 94°C for 30 s, 68°C for 30 s, 72°C for 3 min, and then a final extension at 72°C for 7 min. The nested primers NGSP1 (CGGCTGGATGAACCTCTGGCACG) and NGSP2 (ACATGCGTCGGAGGATCAAGAAG) were used for generating good RACE products of 5′- and 3′-UTR using the same PCR programs.

The DNA products were gel-purified according to the instructions of the kit. Then purified DNA was cloned into pMD19-T vector (TaKaRa) and transformed into* E. coli* Top 10. The positive clones were identified by PCR and sequencing. The sequences of BmLeb5 were assembled with the obtained fragments from 5′RACE and 3′RACE using DNASTAR software and analyzed using the National Center for Biotechnology Information (NCBI) BLAST algorithm.

### 2.5. Bioinformatic Analysis of* BmLeb5*

*BmLeb5* sequence obtained was analyzed using the SeqBuilder program in DNASTAR software package. The signal peptide was predicted with SignalP 4.1 (http://www.cbs.dtu.dk/services/SignalP/). The potential O-glycosylation sites were predicted with NetOGlyc 4.0 (http://www.cbs.dtu.dk/services/NetOGlyc/). The homologous sequences were found by BLASTp (https://blast.ncbi.nlm.nih.gov/Blast.cgi). Multiple protein sequences were aligned using Clustalx 2.1 software. The phylogenetic tree was constructed in MEGA 7 [32] using neighbor-joining method [33].

### 2.6. Quantitative Real-Time PCR (qRT-PCR)

The relative expression levels of BmLeb5 were detected between control and BbHN6-infected silkworm using qRT-PCR. Total RNAs were extracted, respectively, from third instar whole larvae of both the control and the BbHN6-infected groups at 9, 12, 15, 18, 21, 24, 27, 30, 33, 36, 39, 42, and 45 hpi by using a RNApure total RNA rapid extraction kit (Beijing Bolingkewei Biotechnology Co. Ltd.). After being quantified by using a Biophotometer (Eppendorf), the same amounts of total RNAs were prepared for synthesizing the first strand cDNAs using PrimeScript™ RT Reagent Kit (TaKaRa). Then total RNAs of different tissues (cuticle, fat body, hemolymph, Malpighian tubule, midgut, and silk gland) from ten normal larvae of fifth instar were also extracted, respectively, and the cDNA templates were synthesized using the same methods. The relative expression levels of BmLeb5 in hemolymph of the fifth instar larvae at 8, 12, 16, 20, 24, 30, 36, 42, 48, and 54 hpi were also detected using qRT-PCR for the two groups. But the expression was detected for the corresponding fat body from 20 hpi because it was difficult to collect the fat body at the early period.

The qRT-PCR reaction system was 20 *μ*L: 10 *μ*L 2 × SYBR Premix Ex Taq™, 0.4 *μ*L 50 × ROX Reference Dye, 0.5 *μ*L forward primer LebqF (CGTTTAACCCCAAGCCAATA) and reverse primer LebqR (TGCACTCCGAAATCTTTTGT), and 1 *μ*L diluted cDNA template, adding ddH_2_O to 20 *μ*L. The qRT-PCR reaction program was denaturation at 95°C for 5 min, followed by 45 cycles of 95°C for 15 s, 50°C for 15 s, and 72°C for 40 s. The *β-actin* (NM_001126254.1) was used as internal control by the primers BmactinF (CCGTATGCGAAAGGAAATCA) and BmactinR (TTGGAAGGTAGAGAGGGAGG). The qRT-PCR assays were run on an ABI Prism 7300 Sequence Detection System (Applied Biosystems) according to the protocol of the SYBR Premix Ex Taq Kit (TaKaRa). Each sample was repeated three times. The results were first analyzed with Applied Biosystems 7300 System SDS software RQ Study Application Version 1.4. The relative expression differences of* BmLeb5* were calculated by 2^−ΔΔct^ method [34]. Then the results were subjected to IBM SPSS Statistics 19 to analyze the statistical significance. All data were given in terms of relative mRNA expression as terms of means ± SE.

### 2.7. Construction of Expression Vectors and Expression of Recombinant* BmLeb5*

The ORF fragment of* BmLeb5 *without the predicted signal peptide sequence was amplified using the upstream primer (GGATCCCAGGCTTCGTGCCAGAGG) and the downstream primer (AAGCTTCTATTCTTGGAAAATATC) by gradient PCR. The PCR products were gel-purified and cloned into pMD19-T vector (TaKaRa). After being transformed into* E. coli* Top 10, the positive clones containing pMD19-T-BmLeb5 were identified by PCR and sequencing. Then the fragment of* BmLeb5* from pMD19-T-BmLeb5 plasmid was digested with* Bam *HI and* Hind *III enzymes, gel-purified, and ligated into the vector pET-30a(+) (Novagen). The recombinant expression plasmid pET-30a(+)-BmLeb5 was also confirmed as described above. Then the recombinant plasmid was transformed into the cells of* E. coli* BL21.

The* E. coli *BL21 with pET-30a(+)-BmLeb5 was cultured overnight (about 12 h) in Luria-Bertani (LB) broth containing 30 *μ*g/mL kanamycin. The culture was diluted 1 : 100 into fresh LB containing 30 *μ*g/mL kanamycin and further incubated at 37°C on 220 rpm shaker until OD_600_ reached 0.6. The isopropyl *β*-D-thiogalactoside (IPTG) was added to the culture to a final concentration of 0.2 mM to induce the expression of the recombinant BmLeb5 for 8 h at 11°C or 37°C. Then the bacterial cells were harvested, respectively, by centrifugation at 12000*g* for 5 min and resuspended using 2 × SDS-PAGE loading buffer to analysis the expression by 12% sodium dodecyl sulfate polyacrylamide gel electrophoresis (SDS-PAGE).

### 2.8. Purification of BmLeb5

The induced bacterial cells expressing BmLeb5 from the expanding culture at 11°C were resuspended in 20 mL of Binding Buffer (20 mM Tris-HCl, 0.15 M NaCl, pH 8.0) and subjected to sonication at 400 W for 20 minutes (4 s working, 8 s free) on ice. The ultrasonic mixture was centrifuged at 10,000*g* for 20 min. Then the centrifugation supernatant was loaded onto a Ni-IDA-Sepharose column (Novagen) which had been preequilibrated with Binding Buffer. The column was washed using Binding Buffer and Washing Buffer (20 mM Tris-HCl, 20 mM imidazole, and 0.15 M NaCl, pH 8.0) until the OD_600_ of eluate achieved baseline, respectively. The recombinant protein of BmLeb5 was detached by Elution Buffer (20 mM Tris-HCl, 250 mM imidazole, and 0.15 M NaCl, pH 8.0). The last eluate was dialysed by putting into Spectra/Por (R) Universal Closure (Millipore) overnight (about 14 h) using phosphate buffer saline (PBS, PH 7.4). The purified protein was detected by 12% SDS-PAGE. The concentration of purified BmLeb5 was determined by a Bicinchoninic Acid Kit (Beijing Bolingkewei Biotechnology Co. Ltd.).

### 2.9. Western Blot Analysis

For western blot analysis, 0.1 *μ*g of recombinant protein was added in SDS-PAGE gel. Then nonstained SDS-PAGE gel was transferred to polyvinylidene fluoride membrane by electroblotting. After being blocked in PBST (1 × PBS + 0.1% Tween-20) containing 5% (vol vol^−1^) bovine serum albumin (BSA) for 2 h, the membrane was incubated with mouse anti-His-tag polyclonal antibody in PBST for overnight at 4°C. Then the membrane was washed three times with PBST and incubated with horseradish peroxidase-conjugated anti-mouse IgG for 1 h, followed by immunoreactivity detection with diaminobenzidine (DAB) as a chromogenic substrate.

### 2.10. Mass Spectrum Identification

As the molecular weight of recombinant BmLeb5 was bigger than the prediction, Learning Content Management System (LC-MS) was performed to test the recombinant protein molecular weight on TripleTOF® 6600 (SCIEX) by Analyst TF 1.7 (AB Sciex) software. After confirmation of the expressed protein containing BmLeb5, Peptide Mass Fingerprinting (PMF) was developed to identify that the peptide sequence was correct using Mascot v2.3 software.

## 3. Results and Discussion 

### 3.1. Confirmation of Fungal Infection

**Figure 1:**
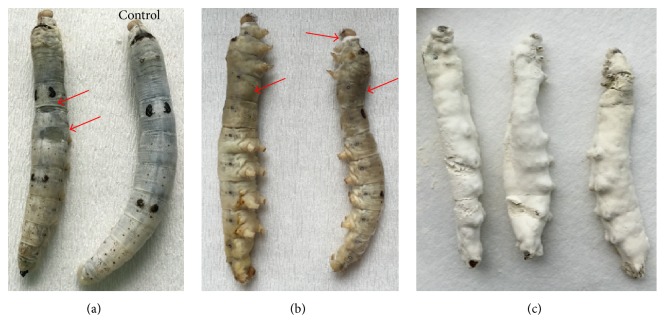
The disease symptoms of fifth instar silkworm infected by* B. bassiana*. (a) The oily spots in the body of diseased silkworm larvae. The control silkworms were kept healthy. (b) The large oily spots on the cuticle and the mycelia growing in cuticle junctions. (c) The mycelia and conidia of* B*.* bassiana* on dead silkworm body.

In this study, all the silkworm larvae were infected with cuticle contact inoculation. The successful infection was first confirmed by the appearance of oily spots on the cuticle as typical symptoms and further observation of the blastospores in hemolymph under microscope (Figures 1(a) and 1(b)). For the third instar silkworm, the typical symptoms appeared about 48 hpi. The same phenomenon also happened to the fifth instar larvae. Finally, all the inoculated larvae died, and 2 days later the cadavers were all rigid and mycosed (Figure 1(c)), while the larvae of the control groups were kept uninfected and cocooned normally.

### 3.2. cDNA Cloning and Bioinformatic Analysis

**Figure 2:**
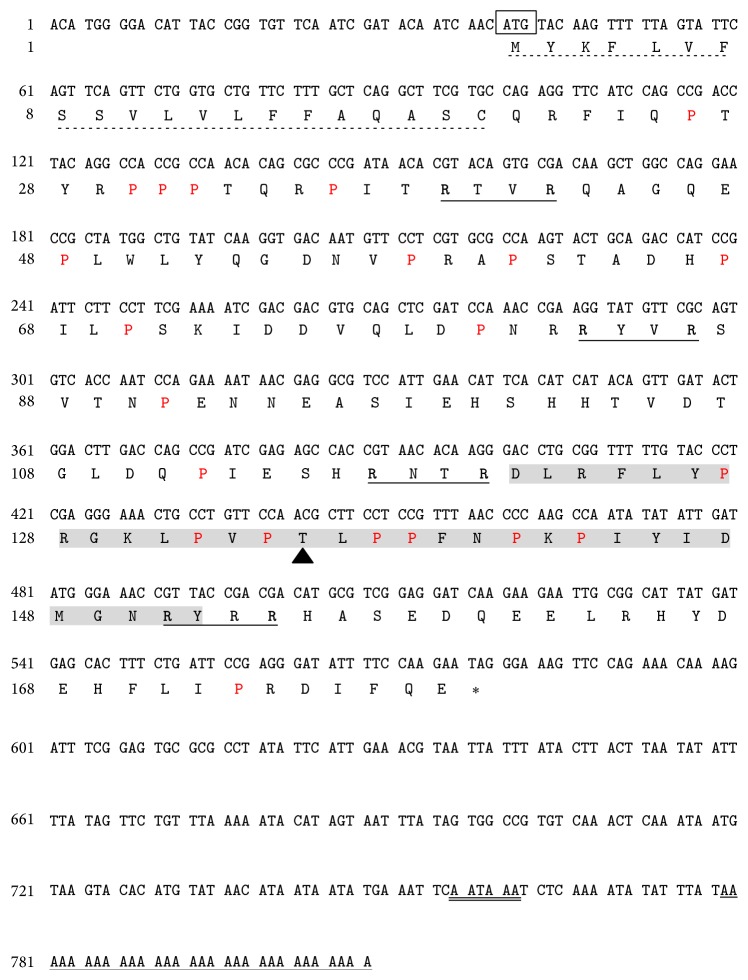
The nucleotide sequence of* BmLeb5 *full-length cDNA and deduced amino acid sequence. The predicted signal sequence at the N-terminus is marked with a dotted underline. The start codon is boxed. The asterisk (*∗*) represents the stop codon. The polyadenylation signal (AATAAA) is marked with double underlines and polyadenylation tail is underlined. The mat peptide of BmLeb5 is marked in gray bars. The O-glycosylation site is indicated by triangle (▲). The proline-rich polypeptides are marked in red. RXXR motifs are marked in bold arginine and underlined. The numeric positions of the nucleotide and amino acid sequences are shown on the left.

The full-length cDNA of* BmLeb5* was obtained by RACE method according to the manufacturer. After sequencing and assembling, the cDNA of* BmLeb5 *was deposited in GenBank (Accession number KX100575). It contained 808 bp consisting of a 39 bp 5′-untranslated region (UTR), a 540 bp ORF, and a 229 bp 3′-UTR (Figure 2). The ORF encodes a proline-rich polypeptide of 179 amino acids with a calculated molecular weight of about 21.1 kDa. The isoelectric point was 7.57 at PH 7.0. SignalP 4.1 server predicted that* BmLeb5 *had a putative N-terminal signal peptide of 20 amino acids, suggesting that it is a secreted protein. Following the signal peptide, there were a putative prosegment of 100 amino acids, a mature peptide of 32 amino acids, and an additional segment of 27 amino acids at the carboxyl terminus (Figure 2). A putative polyadenylation signal of AATAAA was located at 162 nucleotides after translation stop codon and a poly (A) tail was detected in 3′-UTR (Figure 2).

One of the characteristics of* B. mori* lebocins is that they own a unique O-glycosylated threonine residue and the modification seemed to be important for the antimicrobial activity [21, 22]. Synthetic lebocins did not show obvious inhibition zones, probably due to their lack of necessary glycosylations [22, 27]. By NetOGlyc 4.0 Server, an O-glycosylation site was also predicted at Thr^15^ of BmLeb5 mature peptide as in other peptides of lebocins, suggesting that BmLeb5 is also an O-glycosylated antimicrobial peptide and could also play an important role to defend the microbial invasion (Figure 2) [35].

BmLeb5 is also one of the proline-rich AMPs and owns multiple RXXR motifs which could be recognized by intracellular processing enzymes (Figure 2) [36, 37]. There are four RXXR motifs in the prosegment of BmLeb5: R^39^TVR^42^, R^83^YVR^86^, R^117^NTR^120^, and R^151^YRR^154^, following a hydrophilic residue (Figure 2). Most AMPs are synthesized as precursor proteins and then cleaved into some small active peptides to achieve functions. The structure of BmLeb5 implies that the final active peptides of lebocins may be generated from preproteins in the secretory pathway.

**Figure 3:**
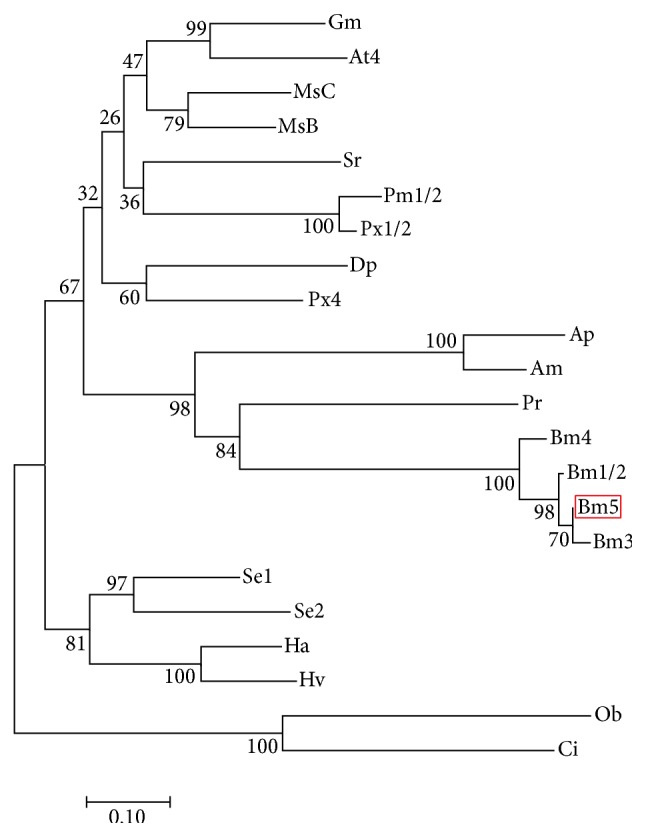
Unrooted phylogenetic tree of BmLeb5 and other homologous proteins. The tree was constructed by the neighbor-joining method within the package MEGA 7. Bootstrap majority consensus values on 1,000 replicates are indicated at each branch point (%). The scale bar represents branch length (number of amino acids substitutions/100 residues). Accession numbers for sequences used in this alignment are as follows (GenBank ID): Bm5 (*Bombyx mori*, KX100575); Bm3 (*Bombyx mori*, NP_001119732.1); Bm4 (*Bombyx mori*, NP_001119731.1); Bm1/2 (*Bombyx mori*, NP_001037468.1); Se1 (*Spodoptera exigua*, AKJ54499.1); Gm (*Galleria mellonella*, ACQ99193.1); Ha (*Helicoverpa armigera*, ALT16900.1); At4 (*Amyelois transitella*, XP_013188814.1); Ap (*Antheraea pernyi*, ACB45567.1); MsB (*Manduca sexta*, ADE20197.1); MsC (*Manduca sexta*, ADE20198.1); Hv (*Heliothis virescens*, ACR78447.1); Pm1/2 (*Papilio machaon*, KPJ06168.1); Px1/2 (*Papilio xuthus*, KPI91575.1); Se2 (*Spodoptera exigua, *AKJ54500.1); Sr (*Samia ricini*, BAD84189.1); Dp (*Danaus plexippus*, EHJ64534.1); Px4 (*Papilio xuthus*, KPI91576.1); Pr (*Pieris rapae*, AEO21919.1); Am (*Antheraea mylitta*, ABG72704.1); Ob (*Operophtera brumata*, KOB78261.1); Ci (*Chrysodeixis includens*, AAS48093.1).

Through the BLASTp search, the results of sequence comparison showed that BmLeb5 was 98% identical to* B. mori *BmLeb3 (NP_001119732.1), 91% identical to* B. mori *BmLeb4 (NP_001119731.1), 96% identical to* B. mori *BmLeb1/2 (NP_001037468.1), 56% identical to* Antheraea mylitta *AmLeb (ABG72704.1) which was the first prorich apidaecin isolated [28], 47% identical to* Pieris rapae *PrLeb (AEO21919.1), and 46% identical to* Antheraea pernyi* ApLeb (ACB45567.1). So far, 5 lebocin genes were identified in the silkworm. Sequence comparison results implied that these lebocin genes had a very close structural relationship and may be evolved from an ancestral gene. Furthermore, the phylogenetic tree was constructed using the amino acid sequences of BmLeb5 homolog proteins. All the lebocin proteins were derived from lepidoptera insects, implying that lebocins were important AMPs in insects (Figure 3). The phylogenetic relationship of silkworm lebocin family showed that an original ancestral gene first divided into two groups, lebocin 4 gene and a common gene for other 3 genes, and then the latter group was duplicated into lebocin 1/2 and lebocin 3 and 5. Lebocin 5 and 3 owned the highest homology and formed a branch (Figure 3). The discovery of BmLeb5 suggested that the lebocin underwent a further gene evolution in silkworm along with environmental threats. The results also suggested that lebocin owned a multiple gene family as cecropins in the silkworm [13, 15, 38].

### 3.3. Expression Profiling of* BmLeb5*

**Figure 4:**
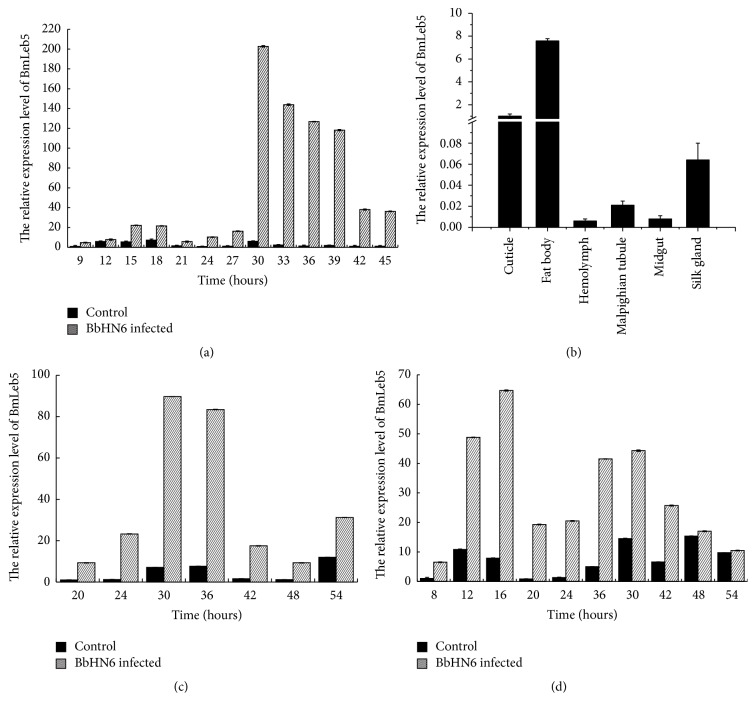
The relative mRNA expression levels of BmLeb5. (a) In the third instar whole larvae of BbHN6-infected and control groups at different times. (b) In different tissues of normal fifth instar larvae. (c) In the fat body of BbHN6-infected and control fifth instar larvae at different times. (d) In the hemolymph of BbHN6-infected and control fifth instar larvae at different times. The *y*-axis indicates the relative expression level of BmLeb5 mRNA transcripts. Vertical bars represent the mean ± SE (*n* = 3).

The relative expression of* BmLeb5 *was detected by qRT-PCR between the control and infected silkworm larvae. In the third instar whole larvae inoculated with BbHN6, the relative expression of* BmLeb5 *was upregulated as compared to the normal ones.* B. bassiana* always could penetrate into silkworm cuticle in 8 hpi. So at the early stage of 9 hpi, the expression of* BmLeb5* had been upregulated about 5-fold than that in the normal groups. Afterwards, it was upregulated about 12-, 13-, 33-, 59-, 95-, 62-, 36-, and 32-fold than that in control ones at 24, 27, 30, 33, 36, 39, 42, and 45 hpi, respectively (Figure 4(a)). The results showed that the expression of* BmLeb5 *was rapidly induced by* B. bassiana* challenge and reached a maximum difference level of 95-fold at 36 hpi. This was consistent with the results of our previous transcriptome analysis [30]. Then, the tissue distribution of* BmLeb5* was further investigated in the fifth instar silkworm larvae with qRT-PCR. The tested tissues included cuticle, fat body, hemolymph, Malpighian tubule, midgut, and silk gland. The* BmLeb5 *mRNA could be detected in all the tested tissues of normal larvae but the highest in the fat body (Figure 4(b)). While the relative expressions of* BmLeb5 *were only upregulated in fat body and hemolymph in the inoculated larvae, no obvious changes were detected in the other four tissues. The maximum upregulation of expression was approximately 20-fold in fat body as compared to the normal ones (Figure 4(c)). Significant difference in hemolymph achieved 22-fold at 20 hpi (Figure 4(d)). When the internal control of *β*-actin was changed to GAPDH, the general trend of expression changes was the same.

Previous studies had demonstrated that* B. mori* lebocin 1/2 could be induced and strongly expressed in fat body by bacterial injection [20].* B. mori* lebocin 3 and lebocin 4 were showed to be induced in the fat body and hemocytes by lipopolysaccharide (LPS) [21]. Our qRT-PCR results for lebocin 5 were consistent with these reports. Without exception, the lebocins are expressed the highest in fat body of silkworm.* B. mori* lebocin 1–3 could be induced by* E. coli *and* B. mori* lebocin 4 could be induced by LPS [20, 22, 29]. Furthermore, the purified peptides of lebocin 1–3 showed antibacterial activity against some Gram-negative bacteria [22]. So far, there are no reports indicating that fungi could induce* B. mori* lebocin expression. Our results suggested that BmLeb5 not only could be induced to the upregulated expression by fungi, but also maybe play an important role in the silkworm to defend* B. bassiana* infection.

### 3.4. Expression and Purification of Recombinant BmLeb5

**Figure 5:**
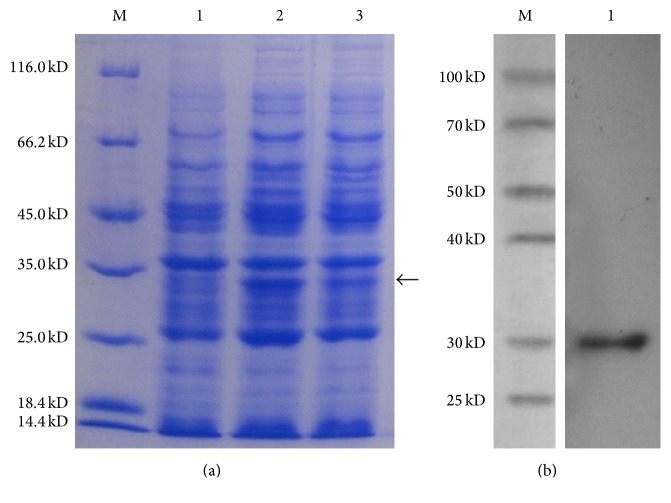
SDS-PAGE and western blot analysis of recombinant BmLeb5. (a) SDS-PAGE analysis of recombinant BmLeb5 expressed at different induction temperatures. Lane M: protein marker (14.4–116 kDa); lane 1: uninduced; lane 2: induced at 11°C; lane 3: induced at 37°C. (b) Western blot analysis of purified recombinant BmLeb5. Lane M: protein marker (25–100 kDa); lane 1: 0.1 *μ*g recombinant protein of BmLeb5.

The recombinant expression vector pET-30a(+)-BmLeb5 was constructed by inserting the BmLeb5 ORF fragment (without signal peptide sequence) and transformed into* E. coli *BL21 for expression of BmLeb5. After IPTG induction at 11°C and 37°C, respectively, the expression quantity of fusion protein was detected by SDS-PAGE. Though the expression was commonly induced at the temperature of 37°C, the induced expression efficiency for BmLeb5 at 11°C was significantly higher than at 37°C (Figure 5(a)). As we know, there are no reports about the successful in vitro expression of any* B. mori* lebocin genes. In* Manduca sexta*, only fusion protein of lebocin B was expressed at 16°C after IPTG induction [27].

After being purified using a Ni-IDA-Sepharose column, the recombinant BmLeb5 exhibited a significant fusion protein band of approximately 32 kDa on 12% SDS-PAGE followed by staining with Coomassie Brilliant Blue R-250. Western blot assay was used to further confirm the purified recombinant protein BmLeb5 using anti-histidine antibody. As shown in Figure 5(b), the single protein band revealed that the prokaryotic expression system achieved an excellent inducible expression of BmLeb5. The expressed recombinant protein would be used for the detection of antifungal activity of BmLeb5 and its antifungal mechanism.

### 3.5. Confirmation of the Recombinant Protein of BmLeb5

**Figure 6:**
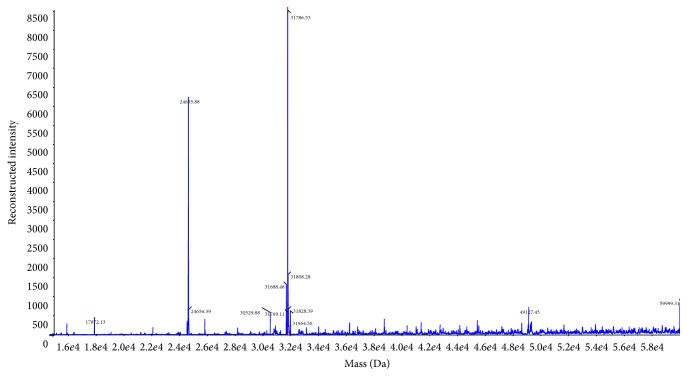
Quality analysis of purified recombinant BmLeb5 with mass spectrometry. There were two substances of 24.6 kDa and 31.8 kDa in the solution of recombinant protein. The 24.6 kDa peak was consistent with the predicted result.

By SDS-PAGE and western blot analysis, the molecular mass of recombinant BmLeb5 was approximately 32 kDa, higher than the predicted 24.6 kDa. In order to confirm the target protein, LC-MS was used to detect the size of molecular mass of the purified recombinant protein. Two peaks of molecular mass, corresponding to 24.6 kDa and 31.8 kDa (Figure 6), were presented. The result could judge that the two substances of 24.6 kDa and 31.8 kDa were main ingredients in the solution of recombinant protein. The 24.6 kDa peak was consistent with the predicted results. After trypsin enzymolysis and secondary mass spectrum identification, the sequences of peptide fragments were blasted with database of UniProt by Mascot v2.3 software. 12 peptides were consistent with the sequence of BmLeb5. After assembling, the vast majority of fragments were completely in accordance with the recombinant protein of BmLeb5. The other peptide of 31.8 kDa was blasted as a protein kinase which probably was reassembled from serine residues of the added tryptase in the solution. Therefore, we established successfully for the first time the expression system for BmLeb5 and the expressed protein could be used for further study of the roles of lebocins both in vitro and in vivo in the silkworm.

In conclusion, we successfully cloned the full-length cDNA of BmLeb5 gene from silkworm using RACE and the sequence was submitted to GenBank. The deduced amino acid sequence contains an O-glycosylation site and four RXXR motifs. The phylogenetic tree suggests that lebocins form a multiple gene family in silkworm. The upregulated expression of BmLeb5 in the whole silkworm larvae, in the fat body, and in hemolymph indicates that it might play an important role in the immune response of silkworm to defend the* B. bassiana* infection. The recombinant protein of BmLeb5 was for the first time successfully expressed with prokaryotic expression system and then purified with Ni-IDA-Sepharose column. We also confirmed the molecular mass and sequences of the expressed protein by LC-MS and PMF. Details of antifungal mechanism of the BmLeb5* in vivo* remain to be explored.
