# Corrigendum to “Molecular Cloning, Bioinformatic Analysis, and Expression of* Bombyx mori* Lebocin 5 Gene Related to* Beauveria bassiana* Infection”

**DOI:** 10.1155/2017/5168354

**Published:** 2017-11-22

**Authors:** Dingding Lü, Chengxiang Hou, Guangxing Qin, Kun Gao, Tian Chen, Xijie Guo

**Affiliations:** ^1^School of Biotechnology and Sericultural Research Institute, Jiangsu University of Science and Technology, Zhenjiang 212018, China; ^2^Nursing School, Zhenjiang College, Zhenjiang 212003, China

In the article titled “Molecular Cloning, Bioinformatic Analysis, and Expression of* Bombyx mori* Lebocin 5 Gene Related to* Beauveria bassiana* Infection” [1], there was an error in Mass Spectrum Identification. The text reading “As the molecular weight of recombinant BmLeb5 was bigger than the prediction, Learning Content Management System (LC-MS) was performed to test the recombinant protein molecular weight on TripleTOF® 6600 (SCIEX) by Analyst TF 1.7 (AB Sciex) software” should be corrected to “As the molecular weight of recombinant BmLeb5 was bigger than the prediction, liquid chromatography-mass spectrometry was performed to test the recombinant protein molecular weight on TripleTOF® 6600 (SCIEX) by Analyst TF 1.7 (AB Sciex) software.”

## References

[1] Lü D., Hou C., Qin G., Gao K., Chen T., Guo X. (2017). Molecular cloning, bioinformatic analysis, and expression of* Bombyx mori* lebocin 5 gene related to* Beauveria bassiana* infection. *BioMed Research International*.

